# Cyclodextrin‐Based Supramolecular Complex Modulates the Bioaccessibility and Vascular Activity of *Plinia peruviana* Extract

**DOI:** 10.1002/open.70266

**Published:** 2026-09-03

**Authors:** Priscila L. Paula, Nubia B. Andrade, Matheus T. Branca, Lucas de Araújo Carvalho, Bruno A. de Oliveira, Adilson D. da Silva, Angela C. Resende, Lara M. Campos, José Maria Barbosa‐Filho, Luiz Fernando C. Oliveira, Vinícius N. Rocha, Ângelo Márcio L. Denadai, Rodrigo L. Fabri

**Affiliations:** ^1^ Bioactive Natural Products Laboratory Department of Biochemistry Institute of Biological Sciences Federal University of Juiz de Fora Juiz de Fora Minas Gerais Brazil; ^2^ Department of Chemistry Institute of Exact Sciences Federal University of Juiz de Fora Juiz de Fora Minas Gerais Brazil; ^3^ Cardiovascular Pharmacology and Medicinal Plants Laboratory Department of Pharmacology and Psychobiology/IBRAG Rio de Janeiro State University Rio de Janeiro Brazil; ^4^ Research Institute for Drugs and Medicines Federal University of Paraíba João Pessoa Paraíba Brazil; ^5^ Center for Spectroscopy and Molecular Structure Department of Chemistry Institute of Exact Sciences Federal University of Juiz de Fora Juiz de Fora Minas Gerais Brazil; ^6^ Department of Veterinary Medicine Faculty of Medicine Federal University of Juiz de Fora Juiz de Fora Minas Gerais Brazil; ^7^ Department of Pharmacy Institute of Life Sciences Federal University of Juiz de Fora Governor Valadares Minas Gerais Brazil

**Keywords:** cyclodextrin, phenolic compounds, *Plinia peruviana*, supramolecular chemistry, vasodilator activity

## Abstract

Cardiovascular diseases remain a major global health challenge, driving the search for plant‐derived bioactive systems with improved pharmaceutical performance. *Plinia peruviana*, a Brazilian native species, is a rich source of phenolic compounds with well‐documented biological activities. However, limitations related to physicochemical instability and low oral bioavailability hinder its practical application. Herein, a supramolecular complex between the ethanolic extract of *P. peruviana* branches and β‐cyclodextrin was developed to improve the biopharmaceutical properties of the extract. Total phenolic content was determined by visible spectrophotometry, while inclusion complex formation was confirmed by nuclear magnetic resonance and Raman spectroscopy. Vasodilatory activity was evaluated through ex vivo vascular reactivity assays using isolated mesenteric arterial beds, and oral safety was assessed in acute and subchronic exposure models following internationally accepted guidelines. In addition, simulated gastrointestinal digestion was employed to investigate phenolic stability and bioaccessibility. The supramolecular complex exhibited biologically relevant vasodilatory activity, showed low oral toxicity, and significantly enhanced the intestinal bioaccessibility of phenolic compounds. Overall, these findings demonstrate that cyclodextrin‐based supramolecular systems represent a promising pharmaceutical delivery strategy for improving the biopharmaceutical performance of phenolic‐rich plant extracts through enhanced intestinal bioaccessibility and a favorable oral safety profile. These characteristics support their further investigation as plant‐based delivery systems for future phytopharmaceutical development for cardiovascular applications.

## Graphical abstract

Cyclodextrin complexation improved the delivery of plant polyphenols by increasing intestinal bioaccessibility while preserving vascular activity and maintaining low oral toxicity. This strategy highlights the potential of supramolecular systems to enhance the therapeutic value of natural products for cardiovascular health.

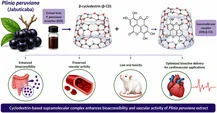

**Abbreviations**
βCD: Beta‐cyclodextrin
ACh: Acetylcholine
ACN: Acetonitrile
ALT: Alanine aminotransferase
ANOVA: Analysis of variance
ANVISA: Brazilian Health Regulatory Agency
AST: Aspartate aminotransferase
CD: Cyclodextrin
CVDs: Cardiovascular diseases
EA: Ellagic acid
EDTA: Ethylenediaminetetraacetic acid
EEB: Ethanolic extract from *Plinia* *peruviana* branches
EEB/βCD: Supramolecular complex from the extract of *Plinia* *peruviana* branches and β‐cyclodextrin
*E*_max_
: Maximal effect
GGT: Gamma‐glutamyl transferase
HE: Hematoxylin‐eosin
HPLC‐DAD: High‐performance liquid chromatography coupled with diode array detection
*ID*_50_
: Inhibitory dose 50%
LD50: Median lethal dose
MAB: Mesenteric arterial bed
NC: Negative control
NE: Norepinephrine
NMR: Nuclear magnetic resonance
OECD: Organisation for Economic Co‐operation and Development
rpm: Rotations per minute
RT: Retention time
UV–vis: Ultraviolet–visible spectroscopy

## 1 Introduction

Cardiovascular diseases continue to be the leading cause of mortality on a global scale [1, 2], constituting a persistent challenge for public health systems, particularly in low‐ and middle‐income countries. While conventional pharmacological therapies are efficacious, their long‐term utilization is frequently associated with deleterious effects, considerable expense, and restricted accessibility. These limitations have given rise to a growing interest in plant‐derived bioactive systems as complementary or alternative strategies for cardiovascular health. Despite extensive preclinical evidence supporting their therapeutic potential, relatively few phytotherapeutic products have progressed toward clinical translation and incorporation into public healthcare systems, underscoring the need for approaches that improve their pharmaceutical performance, physicochemical stability, gastrointestinal behavior, and translational applicability [3, 4].

Species of the genus *Plinia* (Myrtaceae), widely distributed in the Brazilian Atlantic Forest, are recognized as rich sources of phenolic compounds with relevant biological activities [5]. *Plinia peruviana*, commonly known as “jaboticaba,” has been extensively investigated for its antioxidant, antiinflammatory, and cardioprotective properties, which are largely attributed to its complex phytochemical profile [6]. Beyond the edible fruits, nonconventional plant parts such as branches represent underexplored matrices with high phenolic density, particularly tannins and phenolic acids, which have been associated with vascular effects and modulation of cardiovascular risk factors [7, 8, 9, 10, 11].

Despite their promising bioactivity, phenolic‐rich extracts face important physicochemical limitations, including low aqueous solubility, reduced stability under physiological conditions, and variable oral bioaccessibility [9, 12, 13]. These constraints can compromise biological performance and raise concerns regarding dose‐related toxicity, highlighting the importance of comprehensive pharmacological and toxicological evaluation [14]. In this context, supramolecular strategies based on cyclodextrins (CDs) have emerged as powerful tools to modulate the properties of natural bioactives [15]. CDs are cyclic oligosaccharides capable of forming inclusion complexes with structurally diverse compounds, improving solubility, stability, and delivery while potentially reducing adverse effects [16].

The application of CD complexation to multicomponent plant extracts offers an additional level of complexity and opportunity, enabling the development of supramolecular delivery systems capable of improving physicochemical stability, gastrointestinal behavior, and biopharmaceutical performance while maintaining the biological potential of plant‐derived bioactive compounds [17, 18]. However, studies integrating physicochemical characterization, vascular activity, oral safety, and gastrointestinal behavior of such systems remain limited.

Herein, the supramolecular complex formed by the ethanolic extract from *P. peruviana* branches (EEB) and β‐cyclodextrin (βCD) was developed and characterized to investigate its biopharmaceutical properties. The present study aimed to evaluate complex formation, gastrointestinal bioaccessibility, ex vivo vasodilatory activity, and oral safety in order to assess the potential of βCD complexation as a pharmaceutical delivery strategy for phenolic‐rich plant extracts. By integrating physicochemical characterization with pharmacological and toxicological evaluation, this work contributes to the rational development of plant‐based supramolecular delivery systems for future cardiovascular research.

## 2 Experimental Procedures

### 2.1 Extract Preparation

In October 2021, a collection of “jaboticaba” branches (*P. peruviana* (Poir.) Govaerts) was conducted in Belo Horizonte, in the state of Minas Gerais, Brazil. The plant material was identified by Dr. Alexandre Salino, and a voucher specimen (BHCB 174 029) was formally deposited in the BHCB Herbarium at the Federal University of Minas Gerais, under license number A032F41 SISGEN/BRASIL.

The EEB was prepared using the methodology described by Paula et al. (2021) [7]. The plant material underwent a dehydration process at 45 °C for 48 h in a drying oven, followed by grinding in a Wiley‐type knife mill. Subsequently, extraction was performed by static maceration using ethanol as the solvent at room temperature for 15 days, with solvent replacement on alternate days to ensure exhaustive extraction of phytochemical constituents from the plant matrix. The crude ethanolic extract was obtained by solvent removal under reduced pressure using a rotary evaporator (BUCHI Labortechnik AG), with the water bath temperature maintained between 45–55 °C. The crude extract was stored in an airtight glass container under refrigeration at 5 °C.

To ensure reproducibility, all experiments were conducted using a single standardized batch of extract prepared from plant material collected under identical environmental and processing conditions. The phytochemical profile of this extract was previously characterized by high‐performance liquid chromatography (HPLC)‐QTOF‐MS [7].

### 2.2 Preparation of Supramolecular Complex

The EEB/βCD formed by the EEB and βCD was obtained following the procedure outlined by Paula et al. (2023) [18]. It was prepared using the co‐precipitation technique, followed by lyophilization. Initially, 1 g of EEB and 1 g of βCD were weighed. The βCD was then dissolved in 50 mL of distilled water, while the extract was separately dissolved in 50 mL of methanol until fully dissolved. The extract solution was carefully combined with the βCD solutions under constant stirring. The complex solution was kept agitated for 24 h at room temperature and protected from light. Subsequently, this solution was concentrated using a rotary evaporator at 50 °C. The resulting sample was frozen at −80 °C, then lyophilized, and finally stored in an airtight glass jar, protected from light.

### 2.3 Determination of the Total Phenolic and Tannins Assay

The quantification of total phenolic compounds was carried out using visible spectrophotometry, based on adaptations of the Folin–Ciocalteu method [19, 20]. A calibration curve was generated using tannic acid as the standard. For the EEB/βCD, a methanolic stock solution was prepared at a nominal EEB concentration of 1 mg/mL. Absorbance readings were recorded at 770 nm using a UV–vis spectrophotometer, and the resulting calibration curve was defined by the equation *Y* = 0.0415x – 0.1054, with a correlation coefficient of *R* ^2^ = 0.9984. All measurements were performed in triplicate, and data were expressed as mean ± standard deviation. The total phenolic content was reported in milligrams of tannic acid equivalents per gram of extract (mg/g).

Tannin quantification was performed using the gelatin precipitation method, following the protocol outlined in the Brazilian Pharmacopeia [21]. After adding gelatin to the EEB/βCD stock solution, the samples were subjected to centrifugation, and the resulting supernatants were subsequently filtered for further analysis. The resulting filtrates were analyzed to quantify the nonprecipitated phenolics using the Folin–Ciocalteu reagent. The total tannin content was determined by subtracting the amount of nonadsorbed phenols from the total phenol content.

### 2.4 Determination of the Total Flavonoid Assay

The total flavonoid content of the EEB/βCD was determined using a spectrophotometric method adapted from Miliauskas et al. (2004) [22]. Rutin was employed to generate the calibration curve, and the EEB/βCD was solubilized in ethanol at a nominal EEB concentration of 1 mg/mL. Absorbance was measured at 415 nm using a UV–vis spectrophotometer (Thermo Scientific SkanIt Multiskan GO, version 3.2). The calibration curve was defined by the equation *Y* = 0.0199x − 0.0117, with excellent linearity (*R* ^2^ = 0.9995). Analyses were performed in triplicate, and results were expressed as the mean ± standard deviation. Flavonoid content was expressed in milligram equivalents of rutin per gram of extract (mg/g).

### 2.5 Nuclear Magnetic Resonance

The ^1^H‐NMR analysis aimed at evaluating molecular encapsulation was carried out at 25 °C using a Bruker AVANCE III spectrometer (500 MHz). For the assay, EEB, EEB/βCD, and βCD samples were each dissolved in deuterated water (D_2_O, Sigma–Aldrich). The chemical shifts were measured in ppm, referencing the residual water signal, which was fixed at 4.79 ppm.

### 2.6 Raman

Raman spectra were obtained using two complementary techniques. A portable Raman spectrometer (Enwave Optronics Inc.) operating at an excitation wavelength of 532 nm was employed, with laser power adjusted to 20 mW and an exposure time of 20 s per acquisition. The resulting spectrum corresponds to the average of five sequential scans. Additionally, Fourier‐transform Raman (FT‐Raman) spectra were recorded using a Bruker MultiRAM system equipped with a Nd^3+^/YAG laser (1064 nm). For this technique, 512 scans were accumulated with a spectral resolution of 4 cm^−1^ and laser power set to 150 mW. The solid‐state samples analyzed included EEB, EEB/βCD, and βCD. Each spectrum was recorded at least twice to ensure reproducibility and to monitor for any photothermal degradation, based on the consistency of spectral band positions and intensities.

### 2.7 Study In Vitro Digestion

The in vitro simulated digestion protocol was conducted by Brodkorb et al. (2019) [23] in accordance with the INFOGEST 2.0 protocol. The oral phase was conducted at a pH of 7.0 and 37 °C for 2 min using simulated salivary fluid. In the gastric phase, the pH was adjusted to 3.0 using 1 M HCl, and simulated gastric fluid containing pepsin (2000 U/mL; P7000, Sigma–Aldrich) was added to a final volume of 24 mL, followed by incubation at 37 °C for 2 h. In the intestinal phase, the pH was adjusted to 7.0 using a 1 M NaOH solution. The simulated intestinal fluid containing bile salts with sodium deoxycholate and sodium cholate (10 mM; C6750 and C1254, Sigma–Aldrich) and pancreatin (100 U/mL; P7545, Sigma–Aldrich) was added to a final volume of 48 mL. The analyses were conducted in duplicate, and the digested samples were promptly stored at −20 °C.

After centrifugation, supernatants were analyzed for total phenolic content using the Folin–Ciocalteu method [19, 20] with tannic acid as standard. Analyses were performed in triplicate, and results were expressed as mean ± standard deviation (mg TAE g^−1^ dry plant material).

Additionally, the bioaccessible fraction was evaluated as the quantity of phenolic compounds in the soluble fraction after gastric and intestinal phases relative to that in the undigested sample, multiplied by 100, according to the following formula [24]:

Bioaccessibility = Soluble fraction/Undigested sample × 100

### 2.8 Vascular Relaxation

#### 2.8.1 Isolation of the Mesenteric Arterial Bed (MAB)

All experimental procedures were approved by the Ethics Committee for the Care and Use of Experimental Animals of the Roberto Alcantara Gomes Institute of Biology, UERJ (n° ECAU/006/2020). Eleven‐week‐old Wistar rats from the Animal Facility of the Department of Pharmacology and Psychobiology/IBRAG/UERJ were maintained under controlled conditions (23 ± 2 °C; 60 ± 10% relative humidity).

After euthanasia, laparotomy was performed, and the MAB was exteriorized and maintained with modified Krebs solution [25]. The MAB was isolated at its origin from the abdominal aorta and cannulated with a polyethylene tube (PE 50; Clay‐Adams) filled with heparinized Krebs solution.

#### 2.8.2 Measurement of MAB Reactivity to Vasoactive Substances

The MAB isolated was placed in a 10 mL bath and perfused through a cannula connected to a peristaltic pump (MINIPULS 3, Gilson), using Krebs solution at 37 °C, enriched with 95% O_2_ and 5% CO_2_, infused at 4 mL/min. A 30‐min adaptation period was allowed, during which the basal perfusion pressure was maintained between 20 and 40 mmHg [25, 26]. Next, MAB precontraction was initiated by adding norepinephrine (NE, 30 μM) to the solution to maintain perfusion pressure within the range of 80–100 mmHg.

Following the achievement of a pressor response induced by NE, the viability of the vascular endothelium was tested by injecting acetylcholine (ACh, 0.01–300.0 pmol). Subsequently, dose–response curves were conducted (0.1–300.0 μg), in which the response to the vasodilator agent (EEB) was expressed as a percentage reduction of the NE‐induced pressor response. Sodium nitroprusside (SNP, 0.01–300.0 nmol) was used as the positive control. Bolus injections were performed using Hamilton microsyringes (10 and 100 μL) with approximately 5 min intervals between injections, allowing perfusion pressure stabilization. The injections were administered in volumes ranging from 5 to 50 μL. Vasodilatory activity was assessed based on the inhibitory dose 50% (*ID*_50_) and maximal effect (*E*_max_) values obtained from the concentration‐response curves.

### 2.9 Oral Toxicity Study

#### 2.9.1 Acute

Male Swiss mice (30 days old, 25–35 g) were used in the experiments. Animals were obtained from the Center for Reproductive Biology (CRB) at the Federal University of Juiz de Fora (UFJF). Mice were housed in cages under controlled environmental conditions (22 ± 2 °C, 12‐h light/dark cycle) with free access to water and rations. The experimentation procedure was approved by the Ethics Committee on Animal Use (ECAU) of the Vice‐Rectorate for Research/UFJF (protocol n°. 006/2021) and conducted in accordance with applicable national guidelines for animal use and care.

After acclimatization, six mice were randomly divided into six groups: the basal group, which received filtered water; the negative control (NC) group, which received the vehicle (10% Tween 80 in filtered water); the EEB/βCD groups, which received EEB/βCD at nominal EEB concentrations of 250, 500, and 1000 mg/kg; and the βCD group, which received βCD (soluble in filtered water) at a concentration of 1000 mg/kg. Oral administration was performed with 1 mL of the sample per 100 g of animal weight using gavage [27].

Animals were monitored during the first 24 h (0, 15, 30, and 60 min) and subsequently at 4‐h intervals. Afterward, the animals were viewed daily for 14 days after administration to assess weight variations, behavioral parameters, and food and water consumption. At the end of the observation period, all surviving animals were euthanized and autopsied [21].

#### 2.9.2 Subchronic

The subchronic oral toxicity assay was conducted in accordance with the methodologies described by Araújo et al. (2017) [28] and Fabri et al. (2012) [29]. Male Swiss mice (30 days old; 25–35 g) were obtained from the CRB of the UFJF. The animals were kept in standard polypropylene cages under controlled environmental conditions (22 ± 2 °C; 12 h light/dark cycle) with free access to water and rations. All procedures were approved by the ECAU of the Vice‐Rectorate for Research at UFJF (protocol no. 007/2021).

After acclimatization, six mice were randomly divided into six groups: a Basal group received filtered water; a NC group received the vehicle (10% Tween 80 in filtered water); groups were administered the EEB/βCD at nominal EEB concentrations of 50, 100, and 200 mg/kg; and a βCD group received βCD (dissolved in filtered water) at a dose of 200 mg/kg. Oral administration was performed using gavage with 1 mL of the sample per 100 g of body weight [27]. The animals received a daily dose of each sample for 42 days.

Throughout the 42‐day experimental period, animals were individually identified and monitored weekly in order to observe body weight gain. Behavioral changes, biochemical and hematological analyses, and water and food consumption were assessed regularly. At the end of the study, surviving animals were euthanized and subjected to autopsy. Organs presenting macroscopic changes were collected for subsequent histopathological studies according to OECD Guideline for Testing of Chemicals no. 408 (OECD, 2018) and the Brazilian Health Regulatory Agency (ANVISA) guidelines for preclinical safety evaluation [21].

#### 2.9.3 Hematological and Biochemical Analyses

At the end of the experimental period, surviving animals were anesthetized by intraperitoneal injection (i.p.) of xylazine (30 mg/Kg) and ketamine (300 mg/Kg). Blood samples were collected by cardiac puncture, followed by euthanasia. For biochemical analyses (glucose, triglycerides, aspartate aminotransferase [AST], alanine aminotransferase [ALT], gamma‐glutamyl transferase [GGT], total proteins, urea, albumin, creatinine, and bilirubin), blood was centrifuged with ethylenediaminetetraacetic acid (EDTA)‐containing Eppendorf tubes at 3500 rpm for 10 min in order to obtain serum. Hematological parameters (hemoglobin, hematocrit, total leukocyte count, and differential leukocyte count—neutrophils, lymphocytes, monocytes, eosinophils, and basophils) were evaluated using heparinized whole blood. All the analyses were performed using commercial diagnostic kits for each test (Labtest).

#### 2.9.4 Histopathological Analyses

Liver samples collected in the subchronic toxicity study were fixed in 10% buffered formalin. The specimens were processed by dehydration, paraffin‐embedded, and sectioned with a microtome. The sections were stained with hematoxylin‐eosin (HE) for microscopic evaluation captured by the Olympus BX51 microscope, obtaining representative photomicrographs of each group at 40x magnification, employing CellSens Standard software.

### 2.10 Statistical Analysis

Data were expressed as mean ± standard deviation. Statistical analysis was carried out by two‐way analysis of variance (ANOVA), followed by the Bonferroni post hoc test. The *E*_max_ was defined by the highest response amplitude obtained from the dose–effect curves. The ID_50_ was calculated by a nonlinear regression analysis, corresponding to the required dose necessary to achieve 50% of the maximal contraction in response to NE. All the experiments were conducted in triplicate. Statistical significance was established considering *p <* 0.05 or *p <* 0.01. Analysis was performed using GraphPrism 8.0 software.

## 3 Results and Discussion

### 3.1 Phytochemical Content

“Jaboticaba” contains phenolic compounds such as quercetin, rutin, and gallic and ellagic acid (EA) derivatives [7, 8, 11]. Quercetin is recognized for its antioxidant, antiinflammatory, and antiparasitic properties [6, 30, 31]. EA and its derivatives offer potent antioxidant, antimicrobial, antiinflammatory, antiproliferative, and cardioprotective properties [8, 32, 33, 34]. These phenolics are vital for health, preventing disease and mitigating cellular damage related to pathology [7, 35, 36].

In the present study, the quantification of total phenolics, tannins, and flavonoids was performed for the EEB/βCD. As shown in Table 1, the phenolic richness of the extract is confirmed by the high levels of total phenolics. Notably, EEB/βCD exhibited significantly higher tannin content compared to flavonoids (*p <* 0.05), showing the impact of complexation on the extract's phenolic profile.

**TABLE 1:** Quantification of total phytochemical content of EEB/βCD.

Phytochemical content	EEB/βCD, mg/g
Total phenols	319.75 ± 4.24
Tannins	53.07 ± 7.83
Flavonoids	30.15 ± 1.56

*Note:* Values are expressed as mean ± standard deviation in milligrams of tannic acid equivalents per gram of extract (mg/g).

Abbreviations: EEB/βCD, Supramolecular complex from the extract of *Plinia peruviana* branches and β‐cyclodextrin.

HPLC‐QTOF‐MS analysis by Paula et al. (2021) [7] revealed a comprehensive array of tannin derivatives in ethanolic extracts derived from *Plinia cauliflora* leaves (EEL) and branches (EEB). Notably, the branch extract (EEB) showed a considerably higher concentration of these compounds. The EEB's complex phytochemical composition included condensed tannins (e.g., catechin and epigallocatechin) derived from flavan‐3‐ol units, a variety of hydrolyzable tannins (such as EA derivatives, valoneic acid dilactone, casuarictin, tellimagrandin I, and castalagin), and phenolic acids originating from gallic acid. This rich profile underscores the strong pharmacological potential of the branch extracts.

EA and related tannins are well‐recognized for their substantial contribution to antioxidant and antiinflammatory activities [6, 32, 34]. This has led to EA's extensive association with cardioprotective and vasodilatory effects [34, 37, 38]. These cardiovascular benefits are primarily attributed to EA's ability to enhance nitric oxide (NO) bioavailability, modulate endothelial function, and mitigate oxidative stress in vascular tissues. Therefore, the elevated concentration of tannic derivatives present in the EEB may contribute to vascular protection and endothelial relaxation, mechanisms that are likely responsible for the cardiovascular benefits observed in this study.

### 3.2 Nuclear Magnetic Resonance

**FIGURE 1:**
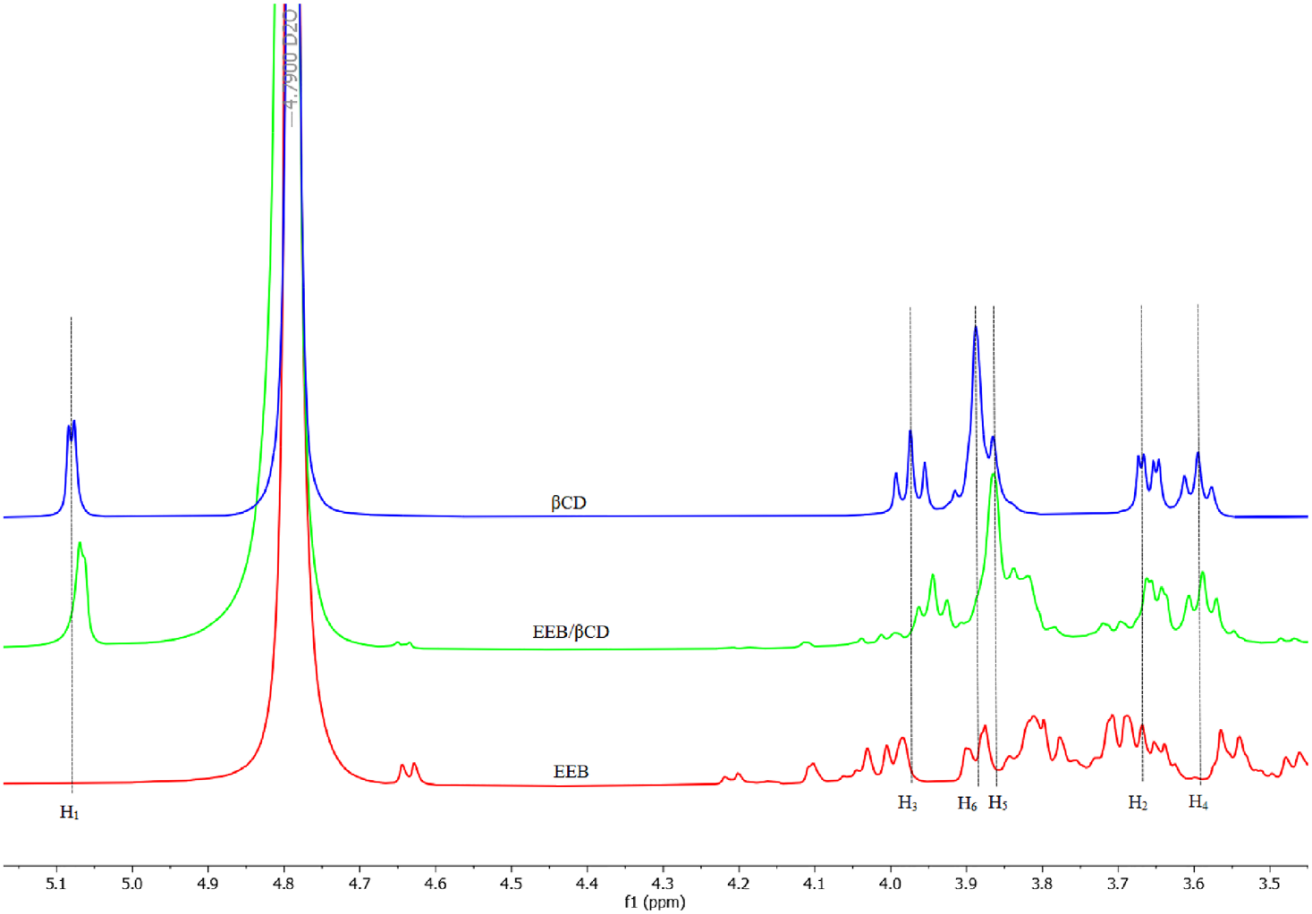
Overlay of the ^1^H‐NMR spectra for the EEB/βCD and βCD with expanded view highlighting signal regions indicative of complex formation.

The characteristic signals of EEB and βCD are present in the ^1^H‐NMR spectrum of the EEB/βCD complex, confirming the effective incorporation of both components after the complexation process. Figure 1 provides an overlay of the ^1^H‐NMR spectra for EEB, EEB/βCD, and βCD, including a magnified view of the region containing these characteristic signals. The complex's spectrum (green) clearly shows the signals corresponding to βCD (blue) and EEB (red).

A comparative analysis of the chemical shift values shown in Table 2 reveals notable changes in the βCD signals upon complex formation, particularly in the protons located within the inner cavity of the CD. The observed chemical shift perturbations follow the order H_5_ > H_3_ > H_6_, which is consistent with the typical inclusion of guest molecules into the hydrophobic cavity of βCD [39]. These results support the formation of supramolecular interactions and partial inclusion of EEB constituents into the βCD cavity.

**TABLE 2:** Chemical shifts for the residual water signal in βCD and EEB/βCD samples.

	βCD, ppm	EEB/βCD, ppm	Δ*δ*, ppm
H_1_	5.0803	5.0606	0.0197
H_2_	3.6599	3.6494	0.0105
H_3_	3.9740	3.9445	0.0295
H_4_	3.5947	3.5887	0.0060
H_5_	3.8698	3.8294	0.0404
H_6_	3.8876	3.8645	0.0231

*Note:* Values are expressed as parts per million (ppm).

Abbreviations: βCD, β‐cyclodextrin; EEB/βCD, Supramolecular complex from the extract of *Plinia peruviana* branches and β‐cyclodextrin.

The supramolecular interaction proposed herein is additionally supported by previously published FTIR data obtained for the same EEB/βCD system, which demonstrated characteristic spectral modifications associated with inclusion complex formation between phenolic constituents and βCD [18].

### 3.3 Raman

**FIGURE 2:**
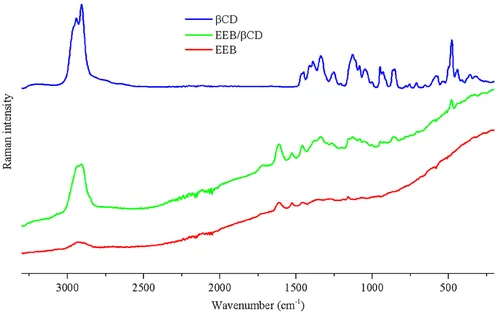
Raman spectra for EEB, EEB/βCD, and βCD.

The interactions between the compounds present in the EEB and βCD were estimated from the Raman spectra. The important regions of the spectra are shown in Figure 2. In the βCD spectrum, the spectral region between 2900 and 2800 cm^−1^, corresponding to the symmetric and asymmetric C—H stretching vibrations of the —CH_2_— and —CH_3_ group, was observed. The band centered at 1140 cm^−1^ corresponds to the C—C stretching vibration, while the bands at 964 and 868 cm^−1^ are attributed to the asymmetric and symmetric stretching vibrations of the C—O—C bonds, respectively [40].

In EEB and EEB/βCD, prominent absorption bands at 2926, 1611, 1526, 1461, and 1158 cm^−1^ were shown. The intensity increases of the bands observed in the EEB/βCD spectrum suggest the formation of the EEB/βCD. In the spectrum of pure EEB, bands at 2926 cm^−1^ can be attributed to the conjugated C—H, 1611 and 1526 cm^−1^ for C=C bonds of aromatic rings, and 1461 and 1158 cm^−1^ corresponding to the stretching vibrations C—O and =C—H of phenol groups and aromatic in‐plane, respectively [41, 42]. It is important to notice that the most predominant bands observed in the Raman spectra of the samples are the ones referring to the presence of phenolic compounds, probably due to stretching aromatic bonds and phenol groups [42, 43]. These spectroscopic findings are in agreement with previously reported FTIR analyses for the EEB/βCD system [18], reinforcing the occurrence of supramolecular interactions between the extract constituents and βCD.

### 3.4 Toxicity Study

The primary stage in investigating a substance's safety profile and toxic potential is the acute toxicity evaluation. This assessment is pivotal in determining the median lethal dose (LD_50_) and other key toxicity indicators. All experiments were conducted in full compliance with the ANVISA's guidelines, specifically Resolution RE no. 90/2004, which outlines the requirements for preclinical toxicity studies of herbal products.

**FIGURE 3:**
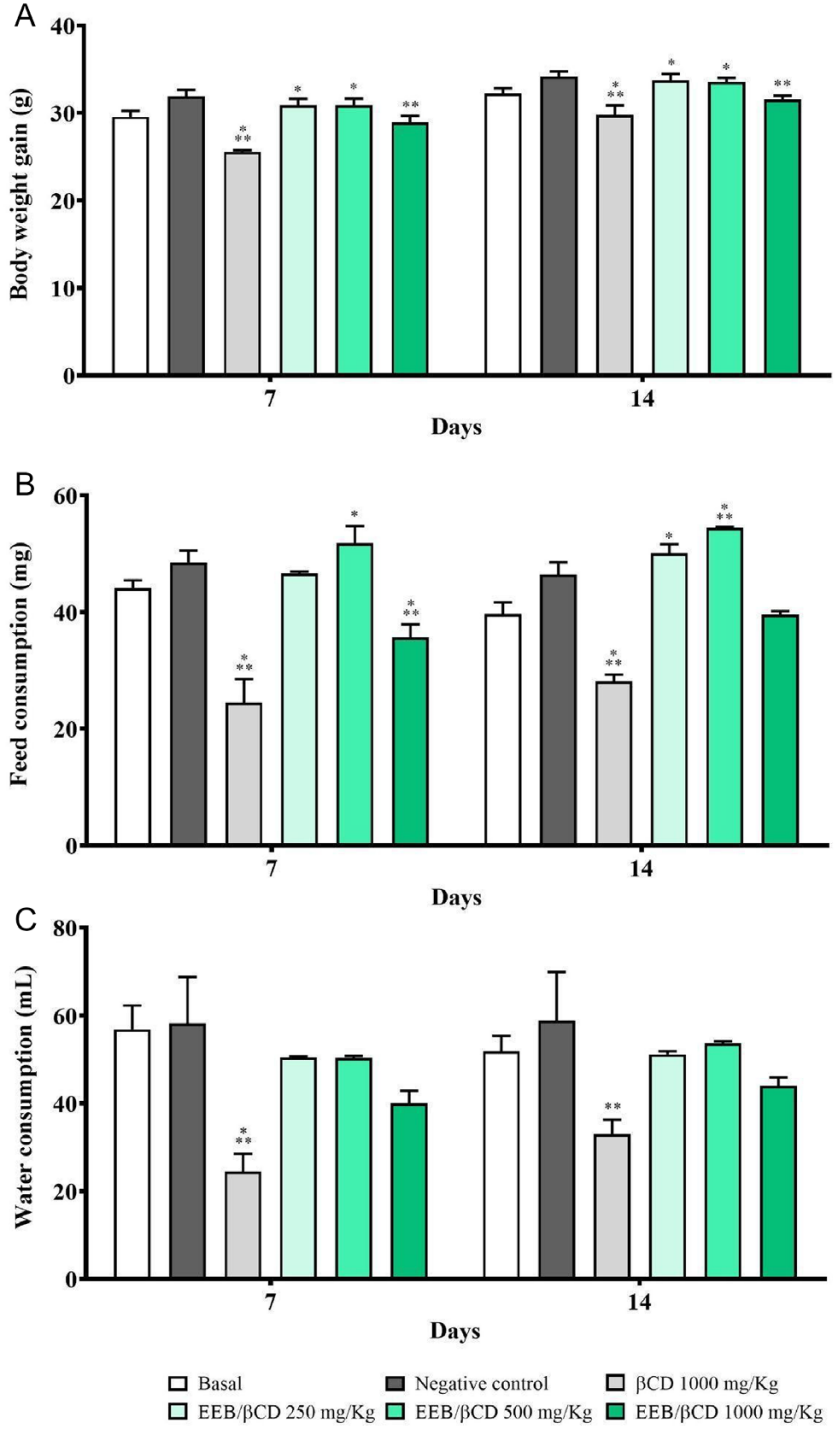
Body weight (A), feed (B), and water (C) consumption during 15 days of treatment with βCD and EEB/βCD. Basal: animal group that received filtered water. NC: Negative control, animal group that received a vehicle (10% Tween 80 in filtered water). βCD: β‐cyclodextrin. EEB/βCD: Supramolecular complex from the extract of *P. peruviana* branches and βCD. *Statistical difference from basal (*p* < 0.05). **Statistical difference from NC (*p* < 0.05). Results were expressed as mean ± standard deviation. One‐way ANOVA was followed by Tukey's multiple comparison post‐hoc test.

In the studies conducted with EEB/βCD, no mortality was observed due to oral administration of the samples during the experimental trial. Furthermore, no significant differences were detected in the body weight gain (Figure 3A), feed and water consumption (Figure 3B,C, respectively), nor were any treatment‐related behavioral alterations observed among groups, indicating adverse effects at the tested doses (*p* > 0.05). The statistical differences observed between groups were attributed to the variability of the animals. The *LD*_50_ of EEB/βCD was greater than 1000 mg/kg. According to the rodent limit, doses limited to 1000 mg/Kg and no deaths exhibit low toxicity [14]. Two animals from the βCD group were excluded due to internal injuries caused during the gavage procedure, which were confirmed after euthanasia and explain the differences observed in the group mean values. These findings were confirmed after necropsy and were considered unrelated to treatment exposure. The reduction in group size likely contributed to the variability observed in body weight and intake parameters.

**FIGURE 4:**
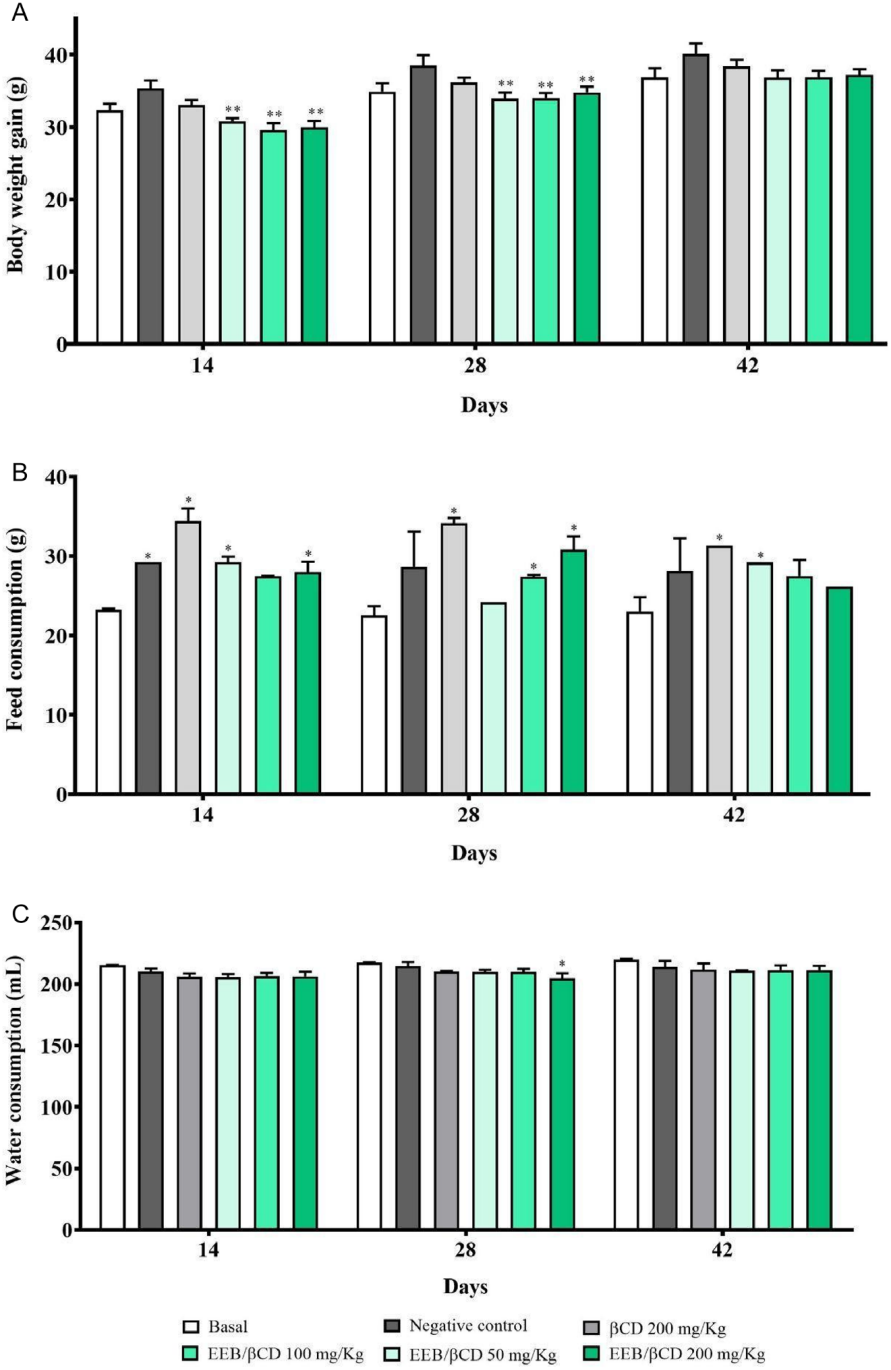
Body weight (A), feed (B), and water (C) consumption during 42 days of treatment with βCD and EEB/βCD. Basal: animal group that received filtered water. NC: Negative control, animal group that received a vehicle (10% Tween 80 in filtered water). βCD: β‐cyclodextrin. EEB/βCD: Supramolecular complex from the extract of *P. peruviana* branches and βCD. *Statistical difference from basal (*p* < 0.05). **Statistical difference from NC (*p* < 0.05). Results were expressed as mean ± standard deviation. One‐way ANOVA was followed by Tukey's multiple comparison post hoc test.

Subchronic toxicity studies allow the characterization of the toxicological profile of samples after repeated administration. This approach permits the identification of potential target organs, toxic effects, and physiological alterations, such as biochemical, hematological, and anatomical parameters. Consequently, the effects of oral administration of EEB/βCD on water consumption, food intake, and body weight gain were monitored daily throughout the period of the test (Figure 4).

Body weight gain in the EEB/βCD group consistently increased throughout the 42‐day experiment (Figure 4A). Notably, by the end of the study, this increase was not statistically different from the NC group (*p* > 0.05). Feed intake showed greater variation in the βCD group, particularly between days 14 and 28 (Figure 4B). Although feed intake for this group decreased by the end of the trial (*p* > 0.05), the increased values observed did not correlate directly with the animals’ average weight gain. Water intake, across all samples, remained stable throughout the entire duration of the trial (Figure 4C).

Table 3 shows the mean body weight and the mean weight of the main organs harvested at the end of the trial. No statistically significant differences were observed between the treated groups and the control groups (*p* > 0.05). Specifically, animals treated with βCD showed an increase in feed intake compared to the basal group, but this was not reflected in body weight gain or the weight of the extracted organs, suggesting that the higher intake did not result in adverse metabolic disturbances and supporting the possibility of individual heterogeneity. Therefore, these results indicate that repeated administration of the EEB/βCD did not compromise the animals’ weight gain, demonstrating the absence of systemic toxic effects.

**TABLE 3:** Average body and major organs weight after the total of days of treatment with βCD and EEB/βCD collected posteuthanasia.

Weight of animals and major organs, g	Basal	NC	βCD 200 mg/Kg	EEB/βCD 50 mg/Kg	EEB/βCD 100 mg/Kg	EEB/βCD 200 mg/Kg
Average weight of the animals	34.71 ± 2.28	36.92 ± 2.26	35.87 ± 2.67	35.36 ± 2.09	35.45 ± 2.07	36.00 ± 1.74
Liver	2.19 ± 0.05^a^	2.04 ± 0.18	2.21 ± 0.08^a^	2.19 ± 0.11^a^	2.06 ± 0.10	2.20 ± 0.09^a^
Spleen	0.22 ± 0.04	0.20 ± 0.03	0.23 ± 0.02	0.21 ± 0.02	0.22 ± 0.03	0.21 ± 0.02
Right kidney	0.27 ± 0.04	0.27 ± 0.04	0.28 ± 0.02	0.24 ± 0.03	0.25 ± 0.04	0.25 ± 0.03
Left kidney	0.28 ± 0.03	0.27 ± 0.05	0.28 ± 0.01	0.25 ± 0.02	0.26 ± 0.02	0.27 ± 0.04

*Note:* Values are expressed as mean ± standard deviation.

Abbreviations: βCD, β‐cyclodextrin; Basal, animal group that received filtered water; NC, negative control, animal group that received a vehicle (10% Tween 80 in filtered water); EEB/βCD, supramolecular complex from the extract of *Plinia* *peruviana* branches and β‐cyclodextrin.

a Statistical difference from EEB/βCD 100 mg/kg (*p < 0.05*). ANOVA followed by a Bonferroni test.

The biochemical parameters are displayed in Table 4. Statistical analysis revealed no significant disparities in the levels of AST, ALT, and total bilirubin between the treated and control groups. These markers are utilized for the evaluation of hepatic and biliary function [29, 44]. Consequently, no hepatotoxicity was detected with any dose during the experimental period, indicating a favorable safety profile. The formation of a complex between the extract and CD has been hypothesized to reduce the toxicological potential of the free extract, thereby supporting the hypothesis that CD may act as a toxicity modulator [45]. This suggests that the administration of these substances at higher concentrations may be safer.

**TABLE 4:** Biochemical parameters in male Swiss mice after treatment with βCD and EEB/βCD.

Biochemical parameters	Basal	NC	βCD 200 mg/kg	EEB/βCD 50 mg/kg	EEB/βCD 100 mg/kg	EEB/βCD 200 mg/kg
Glucose, mg/dL	217.90 ± 2.83	230.01 ± 0.53	224.81 ± 4.75^c^	209.75 ± 1.39^b^	204.51 ± 4.51^a^^,^ b	201.36 ± 1.86^a^^,^ b
Albumin, g/dL	3.40 ± 0.19	3.62 ± 0.37	3.01 ± 0.04	2.78 ± 0.22^b^	2.79 ± 0.15^b^	2.68 ± 0.19^b^
Total protein, g/dL	5.07 ± 0.94	4.73 ± 0.60	6.14 ± 0.06^b^^,^ c	6.03 ± 0.08^c^	5.76 ± 0.29	5.87 ± 0.45^d^
Bilirubin, mg/dL	14.38 ± 1.63	15.18 ± 2.61	13.48 ± 0.53	13.39 ± 1.48	12.85 ± 1.61	11.18 ± 0.17
AST, U/L	3.51 ± 1.28	3.55 ± 2.11	2.46 ± 2.83	2.35 ± 4.44	2.04 ± 4.10	2.61 ± 1.99
ALT, U/L	3.93 ± 0.17	2.75 ± 5.05	4.08 ± 3.73	4.31 ± 2.83	3.77 ± 3.23	4.47 ± 3.44
Urea, mg/dL	69.71 ± 1.47	59.63 ± 1.53^a^	73.05 ± 0.48^b^^,^ c	69.22 ± 0.53^b^^,^ c ^,^ d	61.14 ± 0.36	60.34 ± 2.07
Creatinine, mg/dL	1.86 ± 1.07	0.86 ± 0.003	1.56 ± 0.95	0.60 ± 0.04	0.97 ± 0.03	0.69 ± 0.25
Triglycerides, mg/dL	81.63 ± 0.72	62.47 ± 0.92^a^	84.63 ± 0.58^b^	79.87 ± 3.51 b ^,^ c ^,^ d	89.89 ± 0.38^b^	85.48 ± 2.47^b^
GGT, U/L	4.19 ± 1.99	2.40 ± 2.77	2.04 ± 1.18^c^	3.16 ± 2.77^d^	2.18 ± 1.87^d^	1.10 ± 0.93^a^^,^ b ^,^ c

*Note:* Values are expressed as mean ± standard deviation.

Abbreviations: βCD, β‐cyclodextrin; Basal, animal group that received filtered water; NC, negative control, animal group that received a vehicle (10% Tween 80 in filtered water); EEB/βCD, supramolecular complex from the extract of *Plinia* *peruviana* branches and β‐cyclodextrin.

a Statistical difference from Basal (*p <* 0.05).

b Statistical difference from NC (*p <* 0.05).

c Statistical difference from EEB/βCD 100 mg/Kg (*p <* 0.05).

d Statistical difference from EEB/βCD 200 mg/Kg (*p <* 0.05). ANOVA followed by Bonferroni test.

The protein profile was analyzed by quantifying total proteins and albumin (Table 4). A significant reduction in albumin levels was observed in the groups treated with EEB/βCD 50, 100, and 200 mg/kg compared to the NC (*p <* 0.05). However, total protein levels increased in the groups treated with EEB/βCD 50, 100, and 200 mg/kg and βCD 200 mg/Kg (*p <* 0.05). Although these results may suggest changes in protein parameters, they do not necessarily indicate signs of toxicity, since an increase in total protein levels may be associated with an adaptive response by the body rather than definitive tissue damage [29, 44].

**FIGURE 5:**
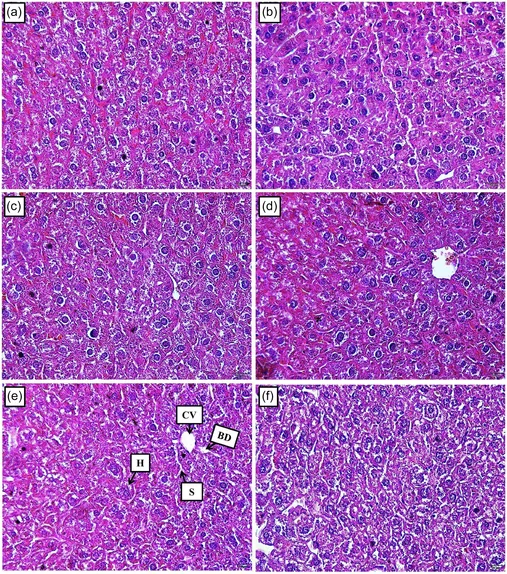
Photomicrographs of the liver tissues from mice in the basal (a), NC (b), βCD (c), EEB/βCD 50 mg/kg (d), and EEB/βCD 100 mg/kg (e), and EEB/βCD 200 mg/kg (f) groups after 42 days of exposure in the subchronic toxicity assay. Sections were stained with HE and observed at 40× magnification. The liver parenchyma in the image shows preserved histoarchitecture, with a visible central vein (CV), bile duct (BD), hepatocytes (H), and sinusoids (S). Basal: animal group that received filtered water. NC: Negative control, animal group that received a vehicle (10% Tween 80 in filtered water). βCD: β‐cyclodextrin. EEB/βCD: Supramolecular complex from the extract of *P. peruviana* branches and βCD.

To confirm if the observed changes were due to liver damage or an adaptive response to prolonged substance administration, histological analysis of the liver was conducted (Figure 5). The analysis of liver tissue from mice treated with EEB/βCD showed an intact hepatic architecture, characterized by organized hepatocyte cords, central nuclei, and basophils, alongside regular sinusoids. Crucially, there was no sign of necrosis, inflammatory infiltrate, or cytoplasmic vacuolization, features indicative of liver damage. This lack of significant morphological change compared to the control group supports the conclusion that the measured increase in total proteins is an adaptive response by the body, rather than hepatotoxicity induced by the treatment.

Evaluation of renal function included measuring blood urea and creatinine levels. While creatinine levels remained statistically similar across all tested groups (*p* > 0.05), significant variations were observed in urea levels (Table 4). Specifically, the groups administered EEB/βCD at 100 and 200 mg/kg exhibited urea levels comparable to those of the basal and NC groups, showing no statistically significant difference (*p <* 0.05). However, elevated urea levels were recorded in the βCD and EEB/βCD 50 mg/kg groups, which were statistically distinct from the NC group (*p <* 0.05).

Urea and creatinine are waste products resulting from protein metabolism and are cleared by the kidneys. Elevated blood concentrations of these compounds signify nitrogen retention, potentially leading to kidney damage. It is important to note that kidney damage is generally indicated by a concurrent rise in both urea and creatinine levels [46, 47, 48].

The lipid profile, specifically serum triglyceride levels, was analyzed as an indicator of coronary artery disease and hyperlipidemia [49]. The groups treated with EEB/βCD at doses of 50, 100, and 200 mg/kg showed triglyceride levels that did not significantly differ from the basal group, yet they were distinct from the βCD and NC groups. Regarding glycemic levels, values were relatively high because the animals were not fasted before euthanasia. Despite this, all groups exhibited comparable blood glucose levels.

GGT is recognized as a marker for liver and biliary diseases, including cholestasis and inflammatory or toxic liver lesions. While sensitive for detecting liver and BD damage, it lacks specificity [49]. Regarding GGT levels in this study, no statistically significant differences (*p* > 0.05) were observed when comparing the basal and NC groups, except for the EEB/βCD 200 mg/kg group. However, all measured values remained within the established normal range for male Swiss mice (0.00–6.00 U/L) [44, 49].

Hematological parameters are vital for toxicity risk assessment, often serving as predictive indicators of adverse effects in humans based on extrapolation from animal models [29]. In this study, the hematological profile remained largely consistent across all experimental groups (*p* > 0.05), suggesting minimal toxicological impact. The only exceptions were hemoglobin levels in the EEB/βCD 50 mg/Kg and βCD groups, which differed when compared to the NC group (Table 5). Crucially, however, these hemoglobin values fell within the established normal reference range for Swiss mice (12.28–14.48 g/dL) [44, 50], indicating the samples did not trigger a toxicity‐related immune response.

**TABLE 5:** Hematological parameters in male Swiss mice after treatment with βCD and EEB/βCD.

Hematological parameters	Basal	NC	βCD 200 mg/kg	EEB/βCD 50 mg/kg	EEB/βCD 100 mg/kg	EEB/βCD 200 mg/kg
Hemoglobin, g/dL	10.57 ± 1.27	8.32 ± 0.83	12.20 ± 0.62^b^^,^ c	12.19 ± 0.12^b^^,^ c ^,^ d	11.60 ± 1.43	11.20 ± 0.01
Hematocrit, %	45.5 ± 0.00	43.42 ± 2.71	43.33 ± 0.94	42.79 ± 0.65	43.83 ± 1.18	41.17 ± 0.71
Total leukocytes, 10^3^ cels/μL	4.23 ± 4.50	3.25 ± 5.66	4.68 ± 2.08	3.76 ± 4.03	5.15 ± 4.58	5.13 ± 3.54
Neutrophil, %	21.25 ± 2.63	18.00 ± 4.24	18.00 ± 5.48	18.00 ± 1.83	21.00 ± 1.00	20.33 ± 1.53
Lymphocyte, %	74.33 ± 5.51	74.67 ± 5.03	72.00 ± 5.83	73.5 ± 3.51	73.67 ± 3.21	72.00 ± 2.83
Eosinophil, %	1.00	0.00	1.33 ± 0.58	1.00 ± 0.00	0.00	0.00
Basophil, %	3.00 ± 2.00	1.00	2.00 ± 0.00	4.50 ± 1.29	2.00 ± 0.82	1.25 ± 0.50
Monocyte, %	2.75 ± 1.50	2.25 ± 1.25	4.25 ± 1.71	4.00 ± 1.41	5.5 ± 0.71^a^	3.67 ± 0.58

*Note:* Values are expressed as mean ± standard deviation.

Abbreviations: Basal, animal group that received filtered water; NC, negative control, animal group that received a vehicle (10% Tween 80 in filtered water); βCD, β‐cyclodextrin; EEB/βCD, supramolecular complex from the extract of *Plinia peruviana* branches and β‐cyclodextrin.

a Statistical difference from Basal (*p* < 0.05).

b Statistical difference from NC (*p* < 0.05).

c Statistical difference from EEB/βCD 100 mg/kg (*p* < 0.05).

d Statistical difference from EEB/βCD 200 mg/kg (*p* < 0.05). ANOVA followed by Bonferroni test.

### 3.5 Digestion Study and Bioaccessibility

The in vitro digestion model was employed to replicate the physiological conditions of the upper gastrointestinal tract, specifically the oral, gastric, and small intestinal phases. Static in vitro digestion models are well established and widely used to predict in vivo digestion outcomes and the gastrointestinal behavior of pharmaceutical and food systems due to their reliability [23]. To assess the impact of the digestive process on the complexed samples, phenolic compound levels were quantified. These values were compared with predigestion measurements after the gastric and intestinal phases, enabling the evaluation of phenolic bioaccessibility.

The relatively low value observed for EEB/βCD during the gastric phase (9.87 ± 0.53) suggests a significant effect of βCD complexation (*p* < 0.05) (Table 6). When compared to the initial phenolic content, the gastric phase results (31.57 ± 2.17 mg/g) indicate a reduced measurable fraction of phenolic compounds under acidic conditions. However, this decrease should not be interpreted solely as degradation, but rather as a consequence of the inclusion complex formation, which limits the release and immediate bioaccessibility of phenolic compounds. The βCD likely acts as a protective carrier, shielding these molecules from direct exposure to the acidic gastric environment and preserving their structural integrity [51, 52, 53]. This behavior is particularly relevant, as phenolic compounds are known to be susceptible to acid‐catalyzed hydrolysis, especially due to the presence of ester and glycosidic bonds in their structures. The cleavage of these bonds under acidic conditions can lead to the formation of simpler compounds, such as phenolic acids and aglycones [50, 54]. Therefore, the reduced phenolic content detected during the gastric phase likely reflects a controlled release effect rather than extensive chemical degradation.

**TABLE 6:** Phenolic content and bioaccessibility of EEB/βCD before and after digestion conditions.

	Total phenolic content, TAE mg/g			Bioaccessibility, %	
	**Before**	**Gastric phase**	**Intestinal phase**	**Gastric**	**Intestinal**
**EEB/βCD**	319.75 ± 4.24	31.57 ± 2.17^b^	242.77 ± 3.69	9.87 ± 0.53^b^	75.93 ± 1.11^a^

*Note:* Values are expressed as mean ± standard deviation.

Abbreviation: EEB/βCD, a supramolecular complex from the extract of *Plinia peruviana* branches and β‐cyclodextrin.

a Statistically significant from before (*p* < 0.05).

b Statistically significant from before (*p* < 0.01). ANOVA followed by Bonferroni.

During the intestinal phase, significantly higher phenolic values were observed compared to the gastric phase (242.77 ± 3.69 mg/g). The increased recovery for EEB/βCD suggests that βCD complexation enhances the release and bioaccessibility of phenolic compounds under intestinal conditions [51, 52]. This behavior can be attributed to the dissociation of the inclusion complex in the more neutral pH environment, facilitating the liberation of previously entrapped phenolics [51, 52, 53]. Additionally, the intestinal conditions may promote the conversion of complex phenolic structures into more soluble and detectable forms, contributing to the higher measured values [55]. These findings indicate that βCD not only protects phenolic compounds during the gastric phase but also enables a controlled release in the intestinal phase, which may favor their subsequent absorption.

These findings are consistent with the well‐established role of CDs as a functional excipient in oral delivery systems [56]. CDs exhibit negligible oral bioavailability, reinforcing their function as molecular carriers rather than active therapeutic agents. Because of their high molecular weight and hydrophilic outer surface, they are not absorbed in the gastrointestinal tract in their intact form [53]. While γ‐CD can be enzymatically hydrolyzed by salivary and pancreatic amylases, natural α‐ and βCDs are resistant to enzymatic digestion but can be partially fermented by the intestinal microbiota. In this context, the ability of βCD to form inclusion complexes plays a crucial role in enhancing the solubility, stability, and controlled release of phenolic compounds, as evidenced by the reduced bioaccessibility during the gastric phase and the increased release under intestinal conditions. Moreover, CDs are widely recognized as safe excipients and are included in major pharmacopoeias, with regulatory approval from agencies such as the Food and Drug Administration and the European Medicines Agency, supporting their broad applicability in oral delivery systems [56].

### 3.6 Vascular Relaxation

Vasodilation is a vital tissue response that maintains homeostasis and tissue integrity through various physiological mechanisms, thus preventing diseases resulting from compromised bodily functions [37]. To test the samples, an intact aortic endothelium was initially contracted using NE until a stable plateau phase was reached. Following this, a concentration–response curve was generated by administering increasing doses of the test samples (0.1–300.00 μg).

**FIGURE 6:**
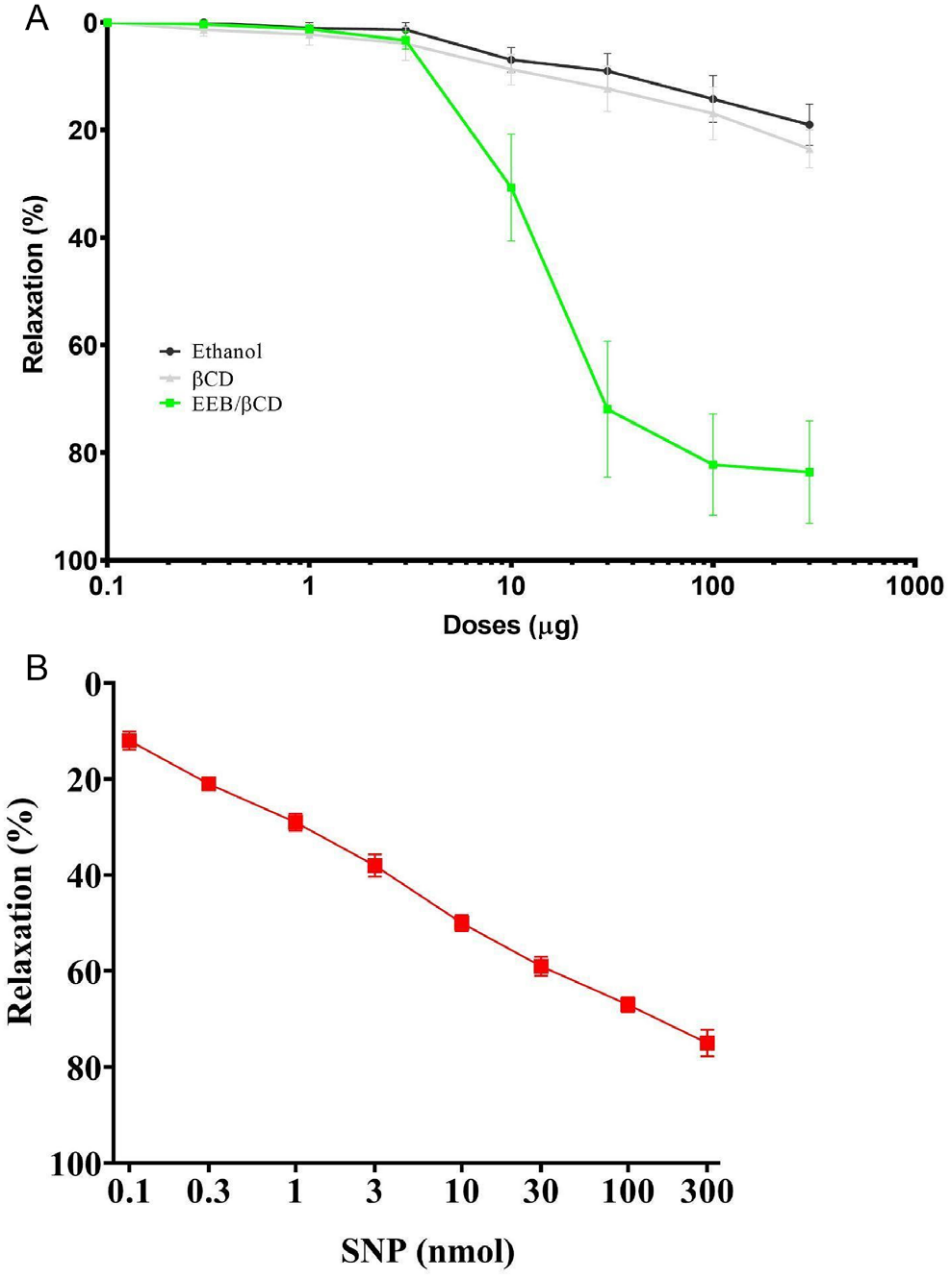
Relaxation percentages of βCD and EEB/βCD (A) and SNP (B) following NE‐induced contraction. βCD: β‐cyclodextrin. EEB/βCD: Supramolecular complex from the extract *P. peruviana* branches and βCD. SNP: Sodium Nitroprusside. Results were expressed as mean ± standard deviation.

Vascular reactivity assays performed in the isolated mouse MAB demonstrated that EEB/βCD induces a clear concentration‐dependent vasodilatory response (Figure 6A). The complex achieved a maximal relaxation (*E*_max_) of 83.6 ± 9.46%, with an ID_50_ of 16.4 ± 2.91 μg, indicating moderate potency and high efficacy in this experimental model. Although less potent than sodium nitroprusside (SNP) (Figure 6B), a well‐established direct nitric oxide (NO) donor [38], which exhibited an ID_50_ of 3.27 ± 0.46 nmol and an *E*_max_ of 75.0 ± 2.73% under the same conditions (Table 7), the EEB/βCD complex demonstrated a greater maximal vasodilatory effect. This distinction highlights the difference between potency and efficacy, as SNP requires lower concentrations to elicit an effect, whereas the complex is capable of producing a more pronounced maximal response [57].

**TABLE 7:** Maximum vasodilatory effect (*E*_max_) and ID_50_ values of βCD, EEB/βCD, and SNP on the relaxation of MABs from rats precontracted with NE.

	Ethanol	βCD	EEB/βCD, μg	SNP, nmol
***E***_**max**_ **, %**	19.0 ± 3.84	23.5 ± 3.46	83.6 ± 9.46^a^	75.0 ± 2.73a
***ID***_**50**_	—	—	16.4 ± 2.91	3.27 ± 0.46
**N**	3	4	4	4

*Note* *:* Values are expressed as mean ± standard deviation.

Abbreviations: βCD, β‐cyclodextrin; EEB/βCD, supramolecular complex from the extract of *Plinia peruviana* branches and β‐cyclodextrin; Ethanol, vehicle used in the experiment; SNP, Sodium Nitroprusside; N, number of animals.

a Statistical difference from vehicle (ethanol) and βCD (*p* < 0.05). ANOVA followed by a Bonferroni test.

The vasodilatory activity observed for EEB/βCD can be attributed to the presence of phenolic compounds, particularly flavonoids and hydrolyzable tannins, previously characterized in this study. These compounds are known to exert cardiovascular protective effects through multiple mechanisms, including antioxidant activity and modulation of endothelial function [10, 58]. In particular, phenolic constituents such as ellagic and gallic acids derivatives have been reported to promote endothelium‐dependent relaxation by enhancing NO bioavailability and activating cyclic nucleotide signaling pathways, including cGMP and cAMP [59]. Additionally, their antioxidant properties may reduce oxidative stress, preventing NO degradation and further contributing to vasorelaxation [6].

On the whole, these findings demonstrate that the EEB/βCD exhibits biologically relevant vasodilatory activity while offering important biopharmaceutical and technological advantages associated with βCD complexation. Although SNP displayed greater pharmacological potency due to its direct nitric oxide donor mechanism, the EEB/βCD complex exhibited high vasodilatory efficacy and demonstrated substantial vasodilatory activity in the ex vivo vascular model.

CD complexation is primarily intended to improve the pharmaceutical performance of natural products rather than to increase their intrinsic pharmacodynamic potency. Accordingly, βCDs are widely recognized as multifunctional pharmaceutical excipients capable of enhancing aqueous compatibility, protecting labile bioactive compounds from degradation, modulating release behavior, and improving the intestinal bioaccessibility of phenolic constituents [15, 16, 17, 18]. In agreement with this, the simulated gastrointestinal digestion assays demonstrated increased bioaccessibility of phenolic compounds after complexation, supporting the role of βCDs as a promising carrier system for plant‐derived bioactives.

This is particularly relevant for phenolic‐rich extracts, whose therapeutic applicability is often limited by low stability, poor solubility, susceptibility to degradation during gastrointestinal transit, and restricted bioavailability after oral administration [15]. Therefore, the vasodilatory activity demonstrated by EEB/βCD, combined with improved bioaccessibility and low oral toxicity, highlights the potential of the EEB/βCD as a bioactive delivery system for cardiovascular applications.

An important aspect to consider is that the enhanced intestinal bioaccessibility observed after simulated digestion could not be expected to translate directly into greater vasodilatory potency in the experimental model employed. The vascular reactivity experiments were performed in an isolated MAB, an ex vivo model that directly exposes the tissue to the tested samples and therefore does not reproduce gastrointestinal absorption, metabolism, or systemic pharmacokinetics. Consequently, the pharmacokinetic advantages expected from CD complexation cannot be fully reflected in this experimental setting.

Furthermore, the vasodilatory response observed for the EEB/βCD should be interpreted considering the complex nature of CD‐phytochemical interactions. CD inclusion is known to occur with different affinities depending on the physicochemical characteristics of individual phytochemical constituents. Consequently, although the EEB/βCD remains rich in phenolic constituents, the relative composition and release kinetics of individual compounds are expected to differ following complexation. These subtle compositional differences represent an inherent feature of multicomponent supramolecular systems and may influence the immediate ex vivo vascular response without compromising the pharmaceutical advantages associated with improved stability, gastrointestinal protection, and intestinal bioaccessibility.

Taken together, these findings support the interpretation that βCD functions primarily as a pharmaceutical carrier capable of improving extract delivery characteristics while providing biologically relevant vasodilatory activity. Whether these improved biopharmaceutical properties translate into enhanced in vivo therapeutic outcomes should be addressed in future in vivo pharmacokinetic and pharmacodynamic studies.

## 4 Conclusion

The present study demonstrates that βCD complexation represents an effective pharmaceutical delivery strategy to improve the biopharmaceutical properties of *P. peruviana* extract while exhibiting biologically relevant vasodilatory activity. The EEB/βCD exhibited enhanced intestinal bioaccessibility together with a favorable oral safety profile, showing no treatment‐related mortality and an estimated LD_50_ greater than 1000 mg/kg following acute exposure, as well as good tolerability after repeated administration. These findings indicate that the principal contribution of βCD in the present system lies in improving extract delivery characteristics rather than altering the intrinsic biological properties of the phytocomplex. Importantly, because vascular activity was evaluated using an isolated MAB preparation, the pharmacokinetic advantages associated with enhanced gastrointestinal bioaccessibility could not be fully represented under the present experimental conditions. Therefore, future in vivo pharmacokinetic and pharmacodynamic studies are warranted to determine whether these improved biopharmaceutical properties translate into enhanced therapeutic performance. The combination of improved bioaccessibility, proven vasodilatory activity, and favorable oral safety supports the continued development of *P. peruviana*‐based supramolecular formulations as promising phytopharmaceutical delivery systems for future cardiovascular research and therapeutic development.

## Author Contributions

P.L.P, R.L.F., A.M.L.D., A.C.R., and L.F.C.O. conceived and designed the experiments. P.L.P, N.B.A., L.R.G., L.A.C., M.R.C., B.A.O. and L.M.C. performed the experiments. P.L.P., L.M.C., V.N.R., B.A.O., and R.L.F. analyzed the data. R.L.F., A.D.S., J.M.B.‐F., A.M.L.D., A.C.R., V.N.R., and L.F.C.O. contributed to the reagents/materials. P.L.P, R.L.F., and A.M.L.D. wrote the paper. All authors have read, edited, and agreed to the published version of the manuscript.

## Funding

This study was supported by grants from the Conselho Nacional de Desenvolvimento Científico e Tecnológico (CNPq, Brazil—grant number: 408700/2021‐1) and Fundação de Amparo à Pesquisa do Estado de Minas Gerais/Brazil (APQ‐01357‐21). Scholarships were provided by the Federal University of Juiz de Fora (UFJF/Brazil and the Coordenação de Aperfeiçoamento de Pessoal de Nível Superior (CAPES, Brazil). The article processing fee for publishing this research was paid by CAPES/Brazil (ROR identifier: 00 × 0ma614). For open access purposes, the authors have assigned the Creative Commons CC BY license to any accepted version of the article.

## Declaration of Generative AI and AI‐Assisted Technologies in the Writing Process

The authors used an artificial intelligence tool (ChatGPT, OpenAI) to support the preparation of the English version of the abstract and keywords and to assist in organizing the list of abbreviations. All outputs were critically reviewed and edited by the authors to ensure scientific accuracy and integrity. The authors accept full responsibility for the content of the manuscript.

## Conflicts of Interest

The authors declare no conflicts of interest.

## Data Availability

All the data from this article is available in the article. The authorization for use of the plant species is registered in SISGEN/Brazil‐A032F41.
